# Molecular Identification of Aminoglycoside-Modifying Enzymes and Plasmid-Mediated Quinolone Resistance Genes among* Klebsiella pneumoniae* Clinical Isolates Recovered from Egyptian Patients

**DOI:** 10.1155/2017/8050432

**Published:** 2017-05-30

**Authors:** Mohamed F. El-Badawy, Wael M. Tawakol, Shaymaa W. El-Far, Ibrahim A. Maghrabi, Saleh A. Al-Ghamdi, Moselhy S. Mansy, Mohammed S. Ashour, Mohamed M. Shohayeb

**Affiliations:** ^1^Department of Microbiology and Immunology, Faculty of Pharmacy, Misr University for Science and Technology, Cairo, Egypt; ^2^Department of Pharmaceutical Microbiology, College of Pharmacy, Taif University, Taif, Saudi Arabia; ^3^General Administration of Pharmaceutical Affairs, Ministry of Health, Alexandria, Egypt; ^4^Department of Clinical Pharmacy, College of Pharmacy, Taif University, Taif, Saudi Arabia; ^5^Department of Microbiology and Immunology, Faculty of Pharmacy, Al-Azhar University, Cairo, Egypt; ^6^Department of Pharmaceutical Microbiology, Faculty of Pharmacy, Tanta University, Tanta, Egypt

## Abstract

Inappropriate use of antibiotics in clinical settings is thought to have led to the global emergence and spread of multidrug-resistant pathogens. The goal of this study was to investigate the prevalence of genes encoding aminoglycoside resistance and plasmid-mediated quinolone resistance among clinical isolates of* Klebsiella pneumoniae*. All* K. pneumoniae* isolates were phenotypically identified using API 20E and then confirmed genotypically through amplification of the specific* K. pneumoniae phoE* gene. All isolates were genotyped by the enterobacterial repetitive intergenic consensus polymerase chain reaction technique (ERIC-PCR). Antibiotic susceptibility testing was done by a modified Kirby-Bauer method and broth microdilution. All resistant or intermediate-resistant isolates to either gentamicin or amikacin were screened for 7 different genes encoding aminoglycoside-modifying enzymes (AMEs). In addition, all resistant or intermediate-resistant isolates to either ciprofloxacin or levofloxacin were screened for 5 genes encoding the quinolone resistance protein (Qnr), 1 gene encoding quinolone-modifying enzyme, and 3 genes encoding quinolone efflux pumps. Biotyping using API 20E revealed 13 different biotypes. Genotyping demonstrated that all isolates were related to 2 main phylogenetic groups. Susceptibility testing revealed that carbapenems and tigecycline were the most effective agents. Investigation of genes encoding AMEs revealed that* acc(6*′*)-Ib* was the most prevalent, followed by* acc(3*′*)-II*,* aph(3*′*)-IV,* and* ant(3*′′*)-I*. Examination of genes encoding Qnr proteins demonstrated that* qnrB* was the most prevalent, followed by* qnrS*,* qnrD,* and* qnrC*. It was found that 61%, 26%, and 12% of quinolone-resistant* K. pneumoniae *isolates harbored* acc(6*′*)-Ib-cr*,* oqxAB,* and* qebA*, respectively. The current study demonstrated a high prevalence of aminoglycoside and quinolone resistance genes among clinical isolates of* K. pneumoniae*.

## 1. Introduction

Few studies have been performed in Egypt concerning the coexistence of genes encoding aminoglycoside-modifying enzymes (AMEs) and plasmid-mediated quinolone resistance (PMQR) among isolates of* Klebsiella pneumoniae*. The present study investigated the prevalence and coexistence of seven genes encoding AMEs and nine genes encoding PMQR. Also, clonal relatedness between* K. pneumoniae* isolates was determined by the enterobacterial repetitive intergenic consensus polymerase chain reaction technique (ERIC-PCR). We found a high prevalence and coexistence of genes encoding quinolone and aminoglycosides resistance that were heterogenous and mostly clonally unrelated.

The most common mechanism of aminoglycoside resistance arises from enzymatic modification rendering aminoglycosides unable to bind with the aminoacyl site of 16S rRNA with a subsequent failure to inhibit protein synthesis [1]. Modification of aminoglycosides is mediated by AMEs, which catalyze the modification at -OH or -NH_2_ groups of the 2-deoxystreptamine nucleus or of the sugar moieties of aminoglycoside molecules [2, 3], resulting in reduced or abolished binding of the aminoglycoside molecule to the ribosome. AMEs can be divided into three families: (i) aminoglycoside N-acetyltransferases (AACs), (ii) aminoglycoside O-phosphotransferases (APHs), and (iii) aminoglycoside O-nucleotidyltransferases (ANTs) [4]. Many of the AMEs result in clinically relevant resistance, but only APHs produce high-level resistance [2].

Regarding the quinolones, there are four known mechanisms of resistance that work separately or in combination, resulting in varying degrees of resistance that range from reduced susceptibility to clinically relevant resistance. These mechanisms may be chromosomal or plasmid-mediated [5]. The term “resistance” in the setting of PMQR refers to any increase in minimum inhibitory concentration (MIC) rather than to an increase above a susceptibility breakpoint [6]. Three mechanisms are responsible for PMQR: (i) target alteration by Qnr, (ii) drug modification by the aminoglycoside acetyltransferase AAC(6′)-Ib-cr, which can reduce ciprofloxacin activity, and (iii) efflux pump activation by two quinolone efflux pumps, which are known as OqxAB and QepA [6, 7].

Qnr proteins protect DNA gyrase and topoisomerase IV from the inhibitory activity of quinolones [8]. Currently, there are six different* qnr* genes:* qnrA*,* qnrB*,* qnrC*,* qnrD*,* qnrS,* and the most recently reported,* qnrVC* [6]. The sequences of* qnr* genes generally differ from each other and from* qnrA* by 35% [9]. Enzymatic inactivation of quinolones arises from aminoglycoside acetyltransferase [AAC (6′)-Ib-cr], which is a bifunctional variant of a common AAC(6′)-Ib. AAC(6′)-Ib-cr acetylates fluoroquinolones, such as ciprofloxacin and norfloxacin, that have an amino nitrogen on the C7 of piperazinyl ring [10]. Finally, we consider PMQR attributed to efflux pumps that specifically extrude quinolones from bacterial cells. Plasmid-mediated quinolone efflux involves two types of pumps, the quinolone efflux pump (QepA) and the olaquindox (OqxAB) efflux pump. QepA belongs to the major facilitator (MFS) family that decreases susceptibility to hydrophilic fluoroquinolones, especially ciprofloxacin and norfloxacin [7]. The* qepA* gene is often located on plasmids that encode aminoglycoside ribosomal methylase* (rmtB)* [6]. The OqxAB pump belongs to the resistance-nodulation-division (RND) family. The OqxAB pump was first detected on a conjugative plasmid (pOLA52) that was harbored by* Escherichia coli* strains isolated from swine manure [7, 11, 12]. The QqxAB efflux pump has wide substrate specificity that includes chloramphenicol, trimethoprim, and quinolones (ciprofloxacin, norfloxacin, and nalidixic acid [13]).* oqxAB* genes are located on plasmids in clinical isolates of* E. coli* and on both chromosomes and plasmids of* Salmonella* spp. and* K. pneumoniae*. We found that* oqxAB* genes are commonly located on the chromosome of* K. pneumoniae *[6, 14].

## 2. Materials and Methods

### 2.1. Bacterial Strains

A total of 114 nonduplicate clinical isolates of* K. pneumoniae *were selected from 301 randomly collected isolates of Gram-negative bacilli. The isolates of* K. pneumoniae* were collected from 84 cases (39 females and 45 males, age between 2 months and 85 years) who were admitted to or attended medical departments at Ain Shams University Hospital, Cairo, Egypt, over a period of one year (May 2012 to April 2013). Isolates of* K. pneumoniae *were recovered from ascitic fluid (*n* = 5), pus (*n* = 2), blood (*n* = 8), throat swab (*n* = 4), endotracheal tube (*n* = 1), sputum (*n* = 25), urinary catheter (*n* = 1), urine (*n* = 22), wound (*n* = 38), cerebrospinal fluid (*n* = 3), central line catheter (*n* = 3), surgical drain (*n* = 1), and nasal swab (*n* = 1).

### 2.2. Isolation and Identification of* K. pneumoniae *Isolates

All isolates of* K. pneumoniae *were initially isolated on MacConkey's agar (Oxoid, UK) and then subcultured on eosin methylene blue (EMB) agar (Scharlau, Spain). The isolated strains were identified phenotypically using API 20E (Biomerieux, France) and then confirmed genotypically through amplification of the specific* phoE *gene using primers and cycling conditions listed in Table 1.

### 2.3. Genotyping of Clinical Isolates

Clonal relatedness between clinical isolates of* K. pneumoniae *was determined by ERIC-PCR. The primer was obtained from Macrogen (Korea, Geumcheon-gu, Seoul). Gene amplification was carried out according to cycling conditions as described in Table 1 using Mastercycler® personal (Eppendorf, California, USA).

### 2.4. Fingerprint Pattern Analysis

The banding pattern generated by ERIC-PCR was analyzed using BioNumerics 7.5 software (Applied Maths, Kortrijk, Belgium). The PCR fingerprint profile was analyzed using Dice (similarity) coefficient. Cluster analysis was performed based on the unweighted pair group method with arithmetic averages (UPGMA) at position tolerance at 0.15, as previously described [15].

### 2.5. Antimicrobial Susceptibility Testing

All* K. pneumoniae *isolates were tested for susceptibility to 23 different antibiotics of several classes. Antimicrobial susceptibility testing was by Kirby-Bauer disc diffusion using Mueller-Hinton agar (MHA) (Oxoid, UK) [16]. Broth microdilution [17] was performed using cation-modified Mueller-Hinton broth (Oxoid, UK) to determine the MIC for the tested antibiotics by the Kirby-Bauer disc-diffusion method. Results were interpreted according to guidelines of the Clinical Laboratory Standards Institute (CLSI) [18]. Both* E. coli* ATCC 25922 and* K. pneumoniae* ATCC 700603 were used as quality-control strains.

### 2.6. Genotypic Detection of Genes Encoding Aminoglycoside and Quinolone Resistance

All* K. pneumoniae* isolates that were resistant to amikacin and/or gentamicin were screened for 7 genes encoding AMEs, namely,* aac(3)-II*,* aac(6*′*)-Ib*,* aac(6*′*)-II*,* ant(3*′′*)-I*,* aph(3*′*)-VI*,* armA, *and* rmtB,* using primers and cycling conditions listed in Table 1.

### 2.7. Genotypic Detection of Genes Encoding Quinolone Resistance

All* K. pneumoniae* isolates that were resistant to ciprofloxacin and/or levofloxacin were screened for 5 quinolone resistance proteins (*qnrA*,* qnrB*,* qnrC*,* qnrD, *and* qnrS*) and one quinolone-modifying enzyme,* acc(6*′*)-Ib-cr*. Also, 3 genes (*oqxA*,* oqxB,* and* qebA*) encoding quinolone efflux pump proteins were screened using primers and cycling conditions listed in Table 2.

### 2.8. Preparation of DNA Templates

DNA was extracted as previously described by Englen and Kelley [19]. Briefly, three to six colonies of bacterial isolates (depending on colony size) were picked from a nutrient agar plate and suspended in 100 *μ*l of DNase-free water in a sterile 1.5 ml microfuge tube to obtain a bacterial suspension equivalent to 1-2 × 10^9^ CFU/ml. The bacterial suspension was placed in a boiling water bath for 10 min to lyse the bacterial cells. The lysed bacterial suspension was centrifuged at maximum speed (13,000 ×g) for 3 min. The supernatant, which contains total genomic DNA, was transferred to a new sterile tube using DNase-free tips. DNA was stored in −20°C.

### 2.9. PCR Setup

The PCR reaction was performed at a final reaction volume of 20 *μ*l. The reaction mixture contained 4 *μ*l of extracted DNA, 4 *μ*l of 5x master mix (HOT FIREPol® Blend Master Mix, Solis BioDyne, Tartu, Estonia), 0.6 *μ*l of forward primer (10 pmol/*μ*l), 0.6 *μ*l of reverse primer (10 pmol/*μ*l), and 10.8 *μ*l distilled water.

## 3. Result

### 3.1. Isolation and Identification

From 301 recovered isolates, 114 (37%) were* K. pneumoniae*, 3 (1%) were* Klebsiella oxytoca*, 61 (20%) were* E. coli*, 48 (16%) were* Proteus *spp., 38 (13%) were* Pseudomonas aeruginosa*, 12 (4%) were* Acinetobacter baumannii*, 9 (3%) were* Serratia marcescens*, 6 (2%) were* Enterobacter cloacae, *and 3 (1%) were single isolates for each of* Providencia stuartii*,* Burkholderia cepacia,* and* Aeromonas hydrophilia*.

### 3.2. Phenotypic and Genotypic Identification of* K. pneumoniae* Isolates

Biotyping of* K. pneumoniae* clinical isolates using API 20E revealed 13 different biotypes. The most prevalent were 5215773 and 5205773, which occurred at a prevalence of 56% (64/114) and 29% (34/114), respectively. Other detected biotypes were 5005573, 5215573, and 5205573, which occurred at a prevalence of 3.5% (4/114), 1.8% (2/114), and 1.8% (2/114), respectively. The lowest detected biotypes were 1205773, 1215773, 5004573, 5204773, 5204553, 5215763, 5217773, and 5214773, which each occurred at a prevalence of 0.88% (1/114).

Genotypic confirmation of phenotypically identified isolates through amplification of the* K. pneumoniae phoE *gene revealed that these isolates were related to* K. pneumoniae*.

**Figure 1:**
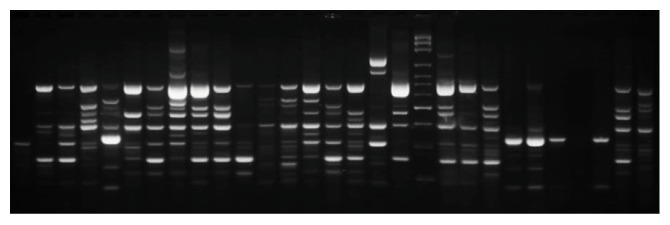
Representative DNA fingerprint pattern of* K. pneumoniae *clinical isolates genotyped by ERIC-PCR.

ERIC-PCR-based DNA fingerprinting identified only 95.6% (109/114) of the* K. pneumoniae *isolates, as 5 isolates gave no band following agarose gel electrophoresis. The 109 genotyped* K. pneumoniae* isolates displayed 85 different fingerprint patterns, as shown in Figure 1.

### 3.3. Fingerprint Pattern Analysis

**Figure 2:**
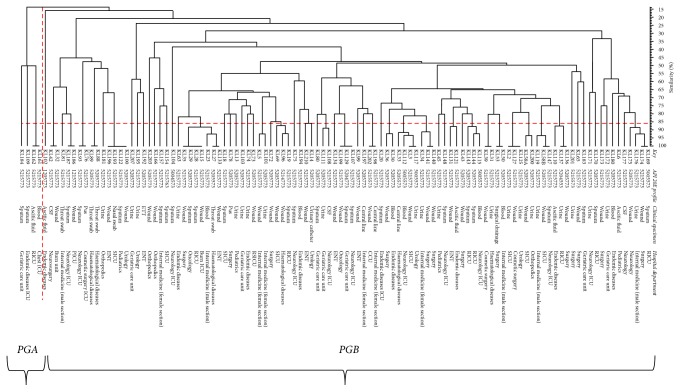
Generated UPGMA dendrogram based on Dice similarity coefficient for clustering of* K. pneumoniae *isolates using ERIC-PCR as DNA fingerprinting method. KL:* Klebsiella pneumoniae*, PGA: phylogenetic group A, and PGB: phylogenetic group B.

A UPGMA dendrogram generated according to Dice (similarity) coefficient revealed that the 85 fingerprint profiles were related to 67 different profiles, including 67 isolates with 18 different combined profiles that included 42 isolates. All genotyped* K. pneumoniae* were classified into 2 major phylogenetic groups (group A and group B), as shown in Figure 2. Phylogenetic group A included 4 isolates (K184, K109, K162, and K161). Two isolates (K161 and K162) within phylogenetic group A had the same fingerprint pattern. Phylogenetic group B contained the remaining 105 isolates.

### 3.4. Correlation between Phenotyping by API 20E and Genotyping by ERIC-PCR

No relationship was found between the phenotypes detected by API 20E and the genotypes detected by ERIC-PCR, as identical clones, such as KL169 and KL174, showed different biotypes (5005773 and 5215773). Other identical genotypes also revealed different biotypes, as shown in Figure 2.

### 3.5. Antimicrobial Susceptibility

Antimicrobial susceptibility tests revealed that carbapenems (imipenem and meropenem) were more effective than 3rd- and 4th-generation cephalosporins for which 75% (86/114) and 75% (85/114) of the isolates were susceptible to imipenem and meropenem, respectively, as shown in Table 3. Regarding non-*β*-lactam antibiotics, tigecycline showed the lowest resistance, as 97% (111/114) of isolates were susceptible. As regarding quinolones and aminoglycosides, we found that 60% (68/114), 26% (30/114), 47% (54/114), and 43% (49/114) of isolates were resistant to gentamicin, amikacin, ciprofloxacin, and levofloxacin, respectively.

Apart from imipenem and meropenem, the MIC_50_ and MIC_90_ values for other tested *β*-lactams ranged between 32 to 512 *μ*g/ml and 256 to >1024 *μ*g/ml, respectively. On the other hand, the MIC_50_ and MIC_90_ for gentamicin and amikacin ranged from ≤0.5 to 8 *μ*g/ml and >1024 *μ*g/ml, respectively, while MIC_50_ and MIC_90_ of ciprofloxacin and levofloxacin ranged from ≤0.5 to 1 *μ*g/ml and 64 to 128 *μ*g/ml, respectively.

### 3.6. Detection of Genes Encoding AMEs

**Figure 3:**
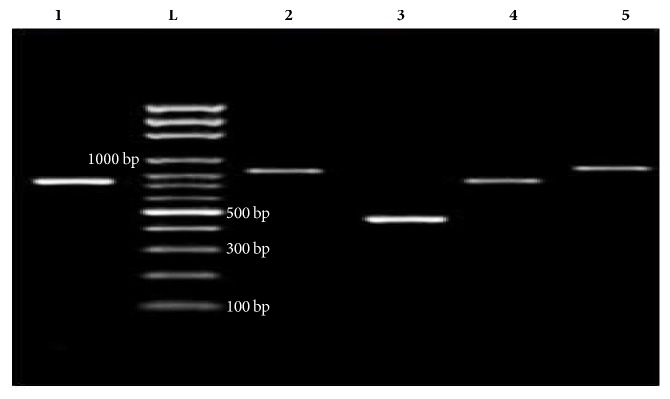
Representative PCR products of detected genes encoding AMEs. Lanes:** (1)*** ant (3*′′*)-I*;** (L)** 100 bp ladder;** (2)*** armA*;** (3)*** acc(6*′*)-Ib*;** (4)*** aph(3*′*)-VI*;** (5)*** acc(3-)-II*.

Genotypic results for AMEs among the aminoglycoside-resistant isolates, as shown in Figure 3, revealed that acetyltransferases were the most prevalent type of AME. The* acc(6*′*)-Ib* and* acc(3*′*)-II* genes were detected among 88% (58/66) and 58% (38/66) of the investigated isolates (Table 4). In contrast, the* acc(6*′*)-II* variant was not detected.

The second most common types of AME were phosphotransferases, followed by nucleotidyltransferases in which* aph(3*′*)-IV* and* ant(3*′*)-I* were detected among 50% (33/66) and 44% (29/66) of isolates, respectively. The lowest detected types of AMEs were ribosomal methylases in which* armA *was detected among 14% (9/66) of tested isolates. A* rmtB* variant was not detected.

### 3.7. Detection of Genes Encoding Qnr Proteins

**Figure 4:**
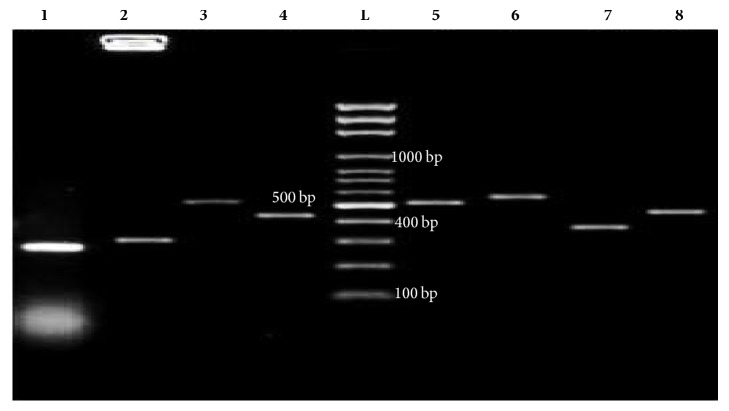
Representative PCR products of detected quinolone resistance genes. Lanes:** (1)*** qnrB*;** (2)*** qnrC*;** (3)*** qnrD*;** (4)*** qnrS*;** (L)** 100 bp ladder;** (5)*** acc(6*′*)-Ib-cr*;** (6)*** oqxA*;** (7) ***oqxb*;** (8)*** qebA.*

Screening of quinolone-resistant isolates for genes encoding Qnr proteins (Figure 4) revealed that* qnrB *was most prevalent (74% (42/57) tested positive). Other detected genes were* qnrS *and* qnrD, *which occurred at a prevalence of 49% (28/57) and 40% (23/57), respectively (Table 4). The gene encoding Qnr protein detected least often was* qnrC*: only one isolate tested positive;* qnrA *was not detected.

### 3.8. Detection of Genes Encoding Quinolone Efflux Pumps

The current study revealed that genes encoding* qebA, oqxA,* and* oqxB* efflux pumps were detected at a prevalence of 12% (7/57), 88% (50/58), and 30% (17/57), respectively, among the quinolone-resistant isolates. Only 26% (15/57) of the isolates harbored both* oqxA *and* oqxB*.

### 3.9. Detection of Gene Encoding Quinolone-Modifying Enzyme

Screening for a quinolone-modifying enzyme among the quinolone-resistant isolates revealed that 61% (35/57) of the tested isolates harbored* acc(6*′*)-Ib-cr*.

### 3.10. Correlation between MIC for Quinolones and Aminoglycosides and Genetic Determinants of Resistance

*K. pneumoniae *isolates that showed elevated MIC (16 to 256 *μ*g/ml) for ciprofloxacin and levofloxacin mainly harbored* qnrB* and* acc(6*′*)-Ib-cr*. Isolates that showed high MIC values (64 to ≥ 1024 *μ*g/ml) for gentamicin and amikacin harbored* aph(3*′*)-VI *and* ant(3*′′*)-I.*

## 4. Discussion 

Aminoglycoside-modifying enzymes are the most important determinants of aminoglycoside resistance among* K. pneumoniae* isolates [28]. The current study revealed that 88% (58/66) and 58% (38/66) of aminoglycoside-resistant* K. pneumoniae* isolates tested positive for* acc(6*′*)-Ib* and* acc(3*′*)-I,I,* respectively. Similar high prevalence rates for* acc(6*′*)-Ib* and* acc(3*′*)-II* among* K. pneumoniae* isolates were reported by Lotfollahi et al. (74% (63/85) and 73% (62/85) for* acc(6*′*)Ib *and* acc(3*′*)-II,* resp., [29]). But lower rates have also been reported (20% (32/162) and 30% (49/162) for* acc(6*′*)-Ib* and* acc (3*′*)-II,* resp., [30]). The present study also found that 44% (29/66) of aminoglycoside-resistant* K. pneumoniae* isolates tested positive for* ant(3*′′*)-1*, which is three times that seen previously (14% (22/162) [30]). For* aph(3*′*)-IV* we found that 50% (33/66) of the aminoglycoside-resistant* K. pneumoniae* isolates harbored this gene, which was similar to the findings of Almaghrabi et al. and Gad et al. (56% (28/50) and 50% (4/8), resp., [28, 31]).

The Qnr proteins are considered as one of the three reported mechanisms of PMQR. The* qnr* genes encode proteins that protect DNA gyrase and topoisomerase IV from inhibition by quinolones and have recently been found worldwide [32]. The current study examined the prevalence of Qnr proteins among* K. pneumoniae* isolates that showed full or intermediate resistance to quinolones. The* qnrA* gene was not detected, which was consistent with Yang et al. [32] but differed from a Portuguese study (19% (4/21) of MDR* K. pneumoniae* isolates [33] and another Egyptian report (12% (14/121) of ESBL-producing* K. pneumoniae* [34]). Regarding* qnrB*, we found that 74% (42/57) of the isolates tested positive, which was slightly more than the 50% (11/22) seen in a Korean study [32]. In contrast, a relatively low prevalence rate was reported by Tunisian study (13% (21/165) [35]). We found that* qnrC *was represented by only a single isolate, which was consistent with findings from the recent Turkish and Tunisian studies that failed to detect* qnrC *among quinolone-resistant* K. pneumoniae* isolates [35, 36]. The current study detected* qnrD* at a prevalence of 40% (23/57); previous work failed to detect this gene [35, 37]. The* qnrS* gene was seen in 49% (28/57) of the investigated* K. pneumoniae* isolates. Lower incidences (9% (2/22) and 2% (3/165)) had been reported in Korean [37] and Tunisian [35] studies, respectively. A much higher incidence (64%, 28/44) was reported in China [38]. We conclude that plasmids carrying* qnr* genes were highly spread in Egypt and China, probably due to misuse of quinolones in clinical settings.

The current work is the first Egyptian study to investigate the QepA and OqxAB efflux pumps among* K. pneumoniae* isolates. We found that 12% (7/57) tested positive for* qepA*, which is far higher than the 2% (5/247) reported previously [39] or the absence of* qebA* among* K. pneumoniae* isolates [40]. The prevalence of* oqxA *and* oqxB* was higher, 88% (50/57) and 30% (17/57), respectively. Previously Rodríguez-Martínez et al. reported values of 76% (87/114) and 75% (86/114), respectively [41]. Only 26% (15/57) of quinolone-resistant* K. pneumoniae* isolates were positive for both* oqxA *and* oqxB*, double that reported earlier (11% (11/102) [32]). Interestingly, Yuan et al. reported that 100% (154/154) of their* K. pneumoniae* isolates tested positive for both* oqxA* and* oqxB*, suggesting that in that case the genes encoding the OqxAB protein were located on the chromosome of* K. pneumoniae*, perhaps as a reservoir for these genes [42]. Thus, high resistance rates to quinolones may be expected among* K. pneumoniae *isolates recovered from clinical settings that frequently prescribe quinolones, since the chromosomal genes coding for OqxAB efflux pump proteins will be overexpressed.

Enzymatic modification of quinolones by the AAC(6′)-Ib-cr enzyme is a third reported mechanism underlying PMQR [32]. The current study revealed that 61% (35/57) of quinolone-resistant* K. pneumoniae* isolates tested positive for* aac(6*′*)-Ib-cr*, which is several times higher than seen by Jlili et al. and by Kim et al. (19% (8/42) and 13% (21/165), resp., [35, 43]).

Genotypic identification of* K. pneumoniae* isolates via amplification of* phoE* identified 81% (92/114) of the* K. pneumoniae* isolates; this finding contradicted Sun et al., who reported 100% [21]. This difference may be due to a mutation in the* phoE *gene of our isolates. Genotyping of* K. pneumoniae* isolates using the ERIC-PCR technique revealed that the majority of isolates had different origins; 32 isolates were related to 18 different single origins, indicating that the spread of* K. pneumoniae* among different hospital departments was due to poor infection control.

## 5. Conclusion 

The current study demonstrated that ACCs were the most prevalent AMEs, followed by APHs and then ANTs. Screening of* qnr* genes revealed that* qnrB* was the most prevalent, followed by* qnrS*. This is the first Egyptian study to detect* qnrC *and* acc(6*′*)-Ib-cr* among quinolone-resistant* K. pneumoniae *isolates. Genotypic identification of* K. pneumoniae* through amplification of the* phoE* gene was not 100%. Most* K. pneumoniae *isolates included in this study displayed different genetic and phenotypic profiles, indicating different origins of dissemination.

## Figures and Tables

**Table 1:** Primer sets and PCR cycling conditions used for genotyping and amplification of genes encoding AMES.

Primer	Sequence	Gene	Reference	Amplification conditions	Amplicon size (bp)
aac(3′)-II	**F**: ATATCGCGATGCATACGCGG **R**: GACGGCCTCTAACCGGAAGG	*aac(3*′*)-II*	[20]	*Initial denaturation* at 95°C for 15 min, then *30 cycles of* 95°C for 1 min, 55°C for 1 min, and 72°C for 5 minutes, and *one cycle* of *final elongation* at 72°C.	877

aac(6′)-Ib	**F**: TTGCGATGCTCTATGAGTGGCTA **R**: CTCGAATGCCTGGCGTGTTT	*aac(6*′*)-Ib*	[20]	*Initial denaturation* at 95°C for 15 min, then *30 cycles of* 95°C for 1 min, 55°C for 1 min, and 72°C for 5 minutes, and *one cycle* of *final elongation* at 72°C.	472
aac(6′)-II	**F**: CGACCATTTCATGTCC **R**: GAAGGCTTGTCGTGTTT	*aac(6*′*)-II*	[20]		542

ant(3′′)-I	**F**: CATCATGAGGGAAGCGGTG **R**: GACTACCTTGGTGATCTCG	*ant(3*′′*)-I*	[20]	*Initial denaturation* at 95°C for 15 min, then *30 cycles of* 95°C for 1 min, 55°C for 1 min, and 72°C for 5 minutes, and *one cycle* of *final elongation* at 72°C.	787

aph(3′)-VI	**F**: ATGGAATTGCCCAATATTATT **R**: TCAATTCAATTCATCAAGTTT	*aph(3*′*)-VI*	[20]	*Initial denaturation* at 95°C for 15 min, then *30 cycles of* 95°C for 1 min, 55°C for 1 min, and 72°C for 5 minutes, and *one cycle* of *final elongation* at 72°C.	780
armA	**F**: CCGAAATGACAGTTCCTATC **R**: GAAAATGAGTGCCTTGGAGG	*armA*	[20]		846

rmtB	**F**: ATGAACATCAACGATGCCCTC **R**: CCTTCTGATTGGCTTATCCA	*rmtB*	[20]	*Initial denaturation* at 95°C for 15 min, then *30 cycles of* 95°C for 1 min, 60°C for 1 min, and 72°C for 5 minutes, and *one cycle* of *final elongation* at 72°C.	769

phoE	**F**: TGGCCCGCGCCCAGGGTTCGAAA **R**: GATGTCGTCATCGTTGATGCCGAG	*phoE*	[21]	*Initial denaturation* at 95°C for 15 min, then *35 cycles of* 95°C for 1 min, 40°C for 1 min, and 72°C for 5 minutes, and *one cycle* of *final elongation* at 72°C.	368
ERIC-1R	**R**: AACCCACGATGTGGGTAGC	—	[22]		—

acc: aminoglycoside acetyl transferase, ant: aminoglycoside nucleotidyl transferase, aph: aminoglycoside phosphor transferase, arm: aminoglycoside ribosomal methylase, and rmt: ribosomal methylase, phoE; phosphoporin E, ERIC, enterobacterial repetitive intergenic consensus.

**Table 2:** Primer sets and PCR cycling conditions used for amplification of genes encoding quinolone resistance.

Prime	Sequence	Gene	Reference	Amplification conditions	Amplicon size (bp)
QnrA	**F**: AGAGGATTTCTCACGCCAGG **R**: TGCCAGGCACAGATCTTGAC	qnrA	[23]	*Initial denaturation* at 95°C for 15 min, then *30 cycles of* 95°C for 1 min, 55°C for 1 min, and 72°C for 5 minutes, and *one cycle* of *final elongation* at 72°C.	580
QnrB	**F**: GGMATHGAAATTCGCCACTG **R**: TTTGCYGYYCGCCAGTCGAA	*qnrB*	[23]		264
QnrC	**F**: GGGTTGTACATTTATTGAATCG **R**: CACCTACCCATTTATTTTCA	*qnrC*	[24]		307

QnrD	**F**: CGAGATCAATTTACGGGGAATA **R**: AACAAGCTGAAGCGCCTG	*qnrD*	[24]	*Initial denaturation* at 95°C for 15 min, then *30 cycles of* 95°C for 1 min, 56°C for 1 min, and 72°C for 5 minutes, and *one cycle* of *final elongation* at 72°C.	533
QnrS	**F**: GCAAGTTCATTGAACAGGGT **R**: TCTAAACCGTCGAGTTCGGCG	*qnrS*	[23]		428
ACC (6′)-Ib-cr	**F**: TTGCGATGCTCTATGAGTGGCTA **R**: CTCGAATGC-CTGGCGTGTTT	*acc (6*′*)-Ib-cr*	[25]		482

OqxA	**F**: CTCGGCGCGATGATGCT **R**: CCACTCTTCACGGGAGACGA	*oqxA*	[26]	*Initial denaturation* at 95°C for 15 min, then *30 cycles of* 95°C for 1 min, 60°C for 1 min, and 72°C for 5 minutes, and *one cycle* of *final elongation* at 72°C.	392

OqxB	**F**: TTCTCCCCCGGCGGGAAGTAC **R**: CTCGGCCATTTTGGCGCGTA	*oqxB*	[26]	*Initial denaturation* at 95°C for 15 min, then *35 cycles of* 95°C for 1 min, 58°C for 1 min, and 72°C for 5 minutes, and *one cycle of final elongation* at 72°C.	512

Qep	**F**: AACTGCTTGAGCCCGTAGAT **R**: GTCTACGCCATGGACCTCAC	*qepA*	[27]	*Initial denaturation* at 95°C for 15 min, then *30 cycles of* 95°C for 1 min, 55°C for 1 min, and 72°C for 5 minutes, and *one cycle* of *final elongation* at 72°C.	596

qnr: quinolone resistance protein, acc(6′)-Ib-cr: aminoglycoside acetyl transferase-ciprofloxacin variant, Oqx: olaquindox, and Qep: quinolone efflux pump.

**Table 3:** Antibiotic susceptibility of *K. pneumoniae* clinical isolates.

Antibiotic	*K. pneumoniae* susceptibility pattern (*n* = 114)			MIC_50_ and MIC_90_	
	Sensitive	Intermediate	Resistant	MIC_50_	MIC_90_
	Number (%)	Number (%)	Number (%)		
Cefotaxime	15 **(13)**	1 **(1)**	98 **(86)**	256	>1024
Ceftazidime	26 **(23)**	14 **(12)**	74 **(65)**	32	>1024
Cefoperazone	17 **(15)**	3 **(2)**	94** (82)**	512	>1024
Cefoperazone/sulbactam	51 **(45)**	12 **(10)**	51 **(45)**	32	1024
Ceftriaxone	18** (16)**	—	96 **(84)**	256	>1024
Cefepime	21 **(18)**	24** (21)**	69** (61)**	32	256
Imipenem	86 **(75)**	5 **(4)**	23 **(20)**	≤0.5	4
Meropenem	85 **(75)**	2 **(2)**	27 **(24)**	≤0.5	16
Tetracycline	32 **(28)**	4 **(3)**	78 **(68)**	128	512
Tigecycline	111 **(97)**	—	3 **(3.0)**	≤0.5	≤0.5
Gentamicin	46 **(40)**	12 **(11)**	56 **(49)**	8	>1024
Amikacin	84 **(74)**	1 **(1)**	29 **(25)**	≤0.5	>1024
Thiamphenicol	7 **(6.0)**	2 **(2)**	105 **(92)**	1024	>1024
Ciprofloxacin	60 **(53)**	3 **(2)**	51 **(45)**	1	128
Levofloxacin	65 **(57)**	5 **(4)**	44 **(39)**	≤0.5	64

**Table 4:** Resistance pattern and genetic profile of quinolone and aminoglycoside resistant *K. pneumoniae *isolates.

Isolate number	Quinolone and aminoglycoside resistance profile	Quinolone resistance genetic profile	Number of quinolone resistance genes	Aminoglycoside resistance genetic profile	Number of aminoglycoside resistance genes	Total number of detected genes
KLP2	CN, AK, CIP	*qnrB*,* qnrD*, *oqxA*, *oqxB*	4	*ant(3*′′*)-I*,* aac(6*′*)-Ib*	2	6
KLP3	CN, CP, LEV	*qnrB*,* qnrD*,* oqxA*	3	*aac(3)-II*,* aac(6*′*)-Ib*	2	5
KLP5	CN	NA	NA	*aac(3)-II*,* aac(6*′*)-Ib*	2	2
KLP6	CN	NA	NA	*aac(3)-II*,* aac(6*′*)-Ib*	2	5
KLP13	CN, AK, CIP, LEV	*qnrB*,* oqxA*, *oqxB*	3	—	—	3
KLP14	CN, AK, CIP, LEV	*qnrB*	1	*aph(3*′*)-VI*, * ant(3*′′*)-I*	2	3
KLP15	CN, AK, CIP	*qnrB*,* oqxA*, *acc(6*′*)-Ib-cr*	3	*ant(3*′′*)-I*,* aac(3)-II*,* aac(6*′*)-Ib*,* aph(3*′*)-VI*,* armA*	5	8
KLP19	CN, AK, CIP, LEV	*qnrB*,* oqxA*, *oqxB*	3	*ant(3*′′*)-I*,* aac(6*′*)-Ib*	2	5
KLP20	CN, AK, CIP, LEV	*qnrB*,* oqxA*	2	*aac(3)-II*,* aac(6*′*)-Ib*	2	4
KLP23	CN, AK, CIP, LEV	*qnrB*,* qnrD*, *qnrS*,* oqxA*	4	*aac(3)-II*,* aac(6*′*)-Ib*,* aph(3*′*)-VI*	3	7
KLP27	CN, CIP, LEV	*qnrB*,* qnrD*,* oqxA*	3	*aac(3)-II*,* aac(6*′*)-Ib*	2	5
KLP28	CN, CIP, LEV	*qnrB*,* oqxA*	2	*aac(6*′*)-Ib*, * aph(3*′*)-VI*,* armA*	3	5
KLP29	CN, AK, CIP	*qnrB*,* oqxA*	2	*ant(3*′′*)-I*, *aac(3)-II*,* aac(6*′*)-Ib*,* aph(3*′*)-VI*	4	6
KLP30	CN, AK, CIP, LEV	*qnrB*, *qnrS*,* oqxA*, *oqxB*, *acc(6*′*)-Ib-cr*	5	*ant(3*′′*)-I*,* aac(3)-II*,* aac(6*′*)-Ib*,* aph(3*′*)-VI*	4	9
KLP33	CN, CIP, LEV	*qnrB*, *qnrS*,* oqxA*, *acc(6*′*)-Ib-cr*	4	*ant(3*′′*)-I*,* aac(3)-II*,* aac(6*′*)-Ib*,* aph(3*′*)-VI*	4	8
KLP34	CN	*oqxA*	1	*aac(3)-II*,* aac(6*′*)-Ib*,* aph(3*′*)-VI*	3	4
KLP35	CN, CIP	*acc(6*′*)-Ib-cr*	1	*aac(6*′*)-Ib*, * aac(6*′*)-Ib*,* aph(3*′*)-VI*	3	4
KLP36	CN, AK, CIP, LEV	*oqxA*	1	*aac(3)-II*,* aac(6*′*)-Ib*	2	3
KLP42	CN	NA	NA	*aac(3)-II*,* aac(6*′*)-Ib*	2	2
KLP43	CN	NA	NA	*aac(3)-II*, * aac(6*′*)-Ib*, *aph(3*′*)-VI*	3	3
KLP50	CN, LEV	*qnrS*,* oqxA*, *acc(6*′*)-Ib-cr*	3	*ant(3*′′*)-I*,* aac(3)-II*,* aac(6*′*)-Ib*	3	6
KLP58A	CIP, LEV	*qnrB*, *qnrS*,* oqxA*, *acc(6*′*)-Ib-cr*	4	NA	NA	4
KLP58B	CN, CIP, LEV	*qnrB*, *qnrS*,* oqxA*, *acc(6*′*)-Ib-cr*	4	*ant(3*′′*)-I*,* aac(3)-II*,* aac(6*′*)-Ib*, *aph(3*′*)-VI*	4	8
KLP63	CN, AK, CIP, LEV	*qnrB*,* oqxA*, *acc(6*′*)-Ib-cr*	3	*ant(3*′′*)-I*,* aac(3)-II*,* aac(6*′*)-Ib*,* aph(3*′*)-VI*	4	7
KLP65	CN	NA	NA	*aac(6*′*)-Ib*	1	1
KLP69	CN, AK, CIP, LEV	*qnrB*,* qnrD*,* oqxA*, *oqxB*, *acc(6*′*)-Ib-cr*	5	—	—	5
KLP72	CN, AK, CIP, LEV	*qnrB*,* qnrD*, *qnrS*,* oqxA*, *acc(6*′*)-Ib-cr*	5	*ant(3*′′*)-I*,* aac(6*′*)-Ib*,* aph(3*′*)-VI*,* armA*	4	9
KLP73	CN	NA	NA	*aac(6*′*)-Ib*	1	1
KLP78	CN, CIP, LEV	*qnrB*,* qnrD*, *oqxA*	3	*aac(3)-II*,* aac(6*′*)-Ib*	2	5
KLP80	CN, AK, CIP, LEV	*qnrD*, *qnrS*,* oqxA*, *acc(6*′*)-Ib-cr*	4	*ant(3*′′*)-I*,* aac(6*′*)-Ib*,* aph(3*′*)-VI*,* armA*	4	8
KLP81	CN, CIP	*qnrB*,* oqxA*, *acc(6*′*)-Ib-cr*	3	*aac(3)-II*,* aac(6*′*)-Ib*,* aph(3*′*)-VI*	3	6
KLP88	CN	NA	NA	*aac(3)-II*, * aac(6*′*)-Ib*,* aph(3*′*)-VI*	3	3
KLP89	CN, AK	NA	NA	*ant(3*′′*)-I*,* aac(3)-II*,* aac(6*′*)-Ib*, * aph(3*′*)-VI*	4	4
KLP93	CN	NA	NA	*ant(3*′′*)-I*,* aac(3)-II*,* aac(6*′*)-Ib*,* aph(3*′*)-VI*	4	4
KLP96	CN, AK, CIP, LEV	*qnrB*,* qnrD*, *qnrS*,* qebA*,* oqxA*, *oqxB*, *acc(6*′*)-Ib-cr*	7	*ant(3*′′*)-I*,* aac(3)-II*,* aac(6*′*)-Ib*,* aph(3*′*)-VI*,* armA*	5	12
KLP99	CN, AK, CIP, LEV	*qnrB*, *oqxA*, *oqxB*, *acc(6*′*)-Ib-cr*	4	*aac(6*′*)-Ib*,* aph(3*′*)-VI*	2	6
KLP100	CN, AK, CIP, LEV	*qnrB*, *oqxA*, *oqxB*, *acc(6*′*)-Ib-cr*	4	*aac(3)-II*,* aac(6*′*)-Ib*	2	6
KLP101	CN, CIP, LEV	*qnrB*, *oqxA*, *acc(6*′*)-Ib-cr*	3	*aac(6*′*)-Ib*, * aph(3*′*)-VI*	2	5
KLP102	CIP, LEV	*qnrD*,* oqxA*	2	NA	NA	2
KLP103	CN, AK, CIP, LEV	*qnrB*, *oqxA*, *oqxB*	3	*aac(6*′*)-Ib*,* aph(3*′*)-VI*	2	5
KLP104	CN, CIP, LEV	*qnrS*,* oqxA*, *acc(6*′*)-Ib-cr*	3	*ant(3*′′*)-I*,* aac(6*′*)-Ib*	2	5
KLP107	CN	NA	NA	*aac(6*′*)-Ib*	1	1
KLP108	CN, LEV	*qnrB*,* qnrS*, *acc(6*′*)-Ib-cr*	3	*aac(3)-II*, * aac(6*′*)-Ib*	2	5
KLP110	CN, CIP, LEV	*qnrB*,* qnrD*, *qnrS*,* oqxA*, *acc(6*′*)-Ib-cr*	5	*ant(3*′′*)-I*,* aac(3)-II*,* aac(6*′*)-Ib*, * aph(3*′*)-VI*	4	9
KLP111	CN	NA	NA	*ant(3*′′*)-I*,* aac(3)-II*,* aph(3*′*)-VI*	3	3
KLP112	CN, CIP, LEV	*qnrB*,* qnrD*,* qnrS*,* oqxA*, *acc(6*′*)-Ib-cr*	5	*ant(3*′′*)-I*,* aac(6*′*)-Ib*,* aph(3*′*)-VI*	3	8
KLP115	CN, CIP, LEV	*qnrB*,* qnrD*,* qnrS*,* oqxA*, *acc(6*′*)-Ib-cr*	5	*aac(6*′*)-Ib*	1	6
KLP117	CIP, LEV	*qnrB*,* qnrD*,* qnrS*,* oqxA*, *acc(6*′*)-Ib-cr*	5	NA	NA	5
KLP119	CN	NA	NA	*aac(3)-II*	1	1
KLP121	CIP, LEV	*qnrB*,* qnrD*,* qnrS*,* oqxA*, *oqxB*	5	NA	NA	5
KLP125	CN, AK, CIP, LEV	*qnrB*,* qebA*,* oqxA*, *oqxB*, *acc(6*′*)-Ib-cr*	5	*aac(6*′*)-Ib*	1	6
KLP127	CN, AK, CIP, LEV	*qnrB*,* qnrD*,* qebA*,* oqxA*, *oqxB*, *acc(6*′*)-Ib-cr*	6	*ant(3*′′*)-I*,* aac(6*′*)-Ib*	2	8
KLP128	CN	NA	NA	—	—	—
KLP129	CN, AK, CIP, LEV	*qnrB*,* qnrS*, *qebA*,* oqxA*, *acc(6*′*)-Ib-cr*	5	*ant(3*′′*)-I*,* aac(6*′*)-Ib*,* aph(3*′*)-VI*,* armA*	4	9
KLP134	CN, AK, CIP, LEV	*qnrB*,* qnrS*,* oqxA*, *oqxB*, *acc(6*′*)-Ib-cr*	5	*ant(3*′′*)-I*,* aac(3)-II*,* aac(6*′*)-Ib*,* aph(3*′*)-VI*	4	9
KLP136	CIP, LEV	*qnrS*, *oqxB*	2	*aac(6*′*)-Ib*	1	3
KLP137	CIP, LEV	*qnrS*,* oqxA*, *acc(6*′*)-Ib-cr*	3	NA	NA	3
KLP140	CN	NA	NA	*aac(3)-II*	1	1
KLP141	CN	NA	NA	*aac(3)-II*	1	1
KLP144	CN, AK, CIP, LEV	*acc(6*′*)-Ib-cr*	1	*ant(3*′′*)-I*,* aac(6*′*)-Ib*,* aph(3*′*)-VI*	3	4
KLP147	CN, LEV	*qnrB*,* qnrD*,* qnrS*,* oqxA*, *acc(6*′*)-Ib-cr*	5	*aac(3)-II*,* aac(6*′*)-Ib*	2	7
KLP148	CN	NA	NA	—	—	—
KLP154	CIP	*qnrB*,* qnrD*,* qnrS*,* oqxA*	4	NA	NA	4
KLP157	CN	NA	NA	*aac(3)-II*, * aac(6*′*)-Ib*,* aph(3*′*)-VI*	3	3
KLP169	CN, AK, CIP, LEV	*qnrB*, *oqxA*, *acc(6*′*)-Ib-cr*	3	*ant(3*′′*)-I*,* aac(3)-II*,* aac(6*′*)-Ib*,* aph(3*′*)-VI*	4	7
KLP170	CN, AK, CIP, LEV	*qnrB*, *oqxA*,* oqxB*, *acc(6*′*)-Ib-cr*	4	*ant(3*′′*)-I*,* aac(6*′*)-Ib*,* aph(3*′*)-VI*	3	7
KLP177	CN, CIP, LEV	—	—	—	—	—
KLP180	CN, AK, CIP, LEV	*qnrB*,* qnrD*,* qnrS*,* oqxA*,* oqxB*, *acc(6*′*)-Ib-cr*	6	*ant(3*′′*)-I*,* aac(6*′*)-Ib*	2	8
KLP184	CIP, LEV	*qnrS*,* oqxA*,* oqxB*	3	NA	NA	3
KLP186	CN, CIP	*qnrC*,* qnrD*, * qnrS*,* oqxA*	4	*aac(6*′*)-Ib*	1	5
KLP187	CIP	*qnrS*,* qnrD*	2	NA	NA	2
KLP197	CN, AK, CIP, LEV	*qnrS*,* qebA*,* oqxA*, *acc(6*′*)-Ib-cr*	4	*ant(3*′′*)-I*,* aac(6*′*)-Ib*,* aph(3*′*)-VI*,* armA*	4	8
KLP198	CN, AK, CIP, LEV	*qnrB*,* qnrD*,* qebA*,* oqxA*, *acc(6*′*)-Ib-cr*	5	*aac(3)-II*,* aac(6*′*)-Ib*	2	7
KLP199	CN	NA	NA	*aac(3)-II*,* aac(6*′*)-Ib*	2	2
KLP201	CN, CIP, LEV	*qnrB*,* qnrD*,* oqxA*	3	*ant(3*′′*)-I*,* aac(3)-II*,* aac(6*′*)-Ib*,* aph(3*′*)-VI*,* armA*	5	8
KLP207	CN, AK, CIP, LEV	*qnrB*,* qnrS*, *qebA*,* oqxA*, *acc(6*′*)-Ib-cr*	5	*ant(3*′′*)-I*,* aac(6*′*)-Ib*, *armA*	3	8

KL: *Klebsiella pneumoniae*, CN: gentamicin, AK: amikacin, CIP: ciprofloxacin, LEV: levofloxacin, and NA: not applicable.
